# Evaluation of the Cardiovascular Effects of Methylmercury Exposures: Current Evidence Supports Development of a Dose–Response Function for Regulatory Benefits Analysis

**DOI:** 10.1289/ehp.1003012

**Published:** 2011-01-10

**Authors:** Henry A. Roman, Tyra L. Walsh, Brent A. Coull, Éric Dewailly, Eliseo Guallar, Dale Hattis, Koenraad Mariën, Joel Schwartz, Alan H. Stern, Jyrki K. Virtanen, Glenn Rice

**Affiliations:** 1 Industrial Economics Inc., Cambridge, Massachusetts, USA; 2 Harvard School of Public Health, Department of Biostatistics, Boston, Massachusetts, USA; 3 Health and Environmental Group, Centre Hospitalier Universitaire de Quebec, Quebec, Canada; 4 Johns Hopkins School of Public Health, Department of Epidemiology, Baltimore, Maryland, USA; 5 National Center for Cardiovascular Research, Department of Cardiovascular Epidemiology and Population Genetics, Madrid, Spain; 6 George Perkins Marsh Institute, Clark University, Worcester, Massachusetts, USA; 7 Office of Environmental Health Assessment, Washington State Department of Health, Olympia, Washington, USA; 8 Harvard School of Public Health, Department of Environmental Health/Department of Epidemiology, Boston, Massachusetts, USA; 9 Independent Consultant, Metuchen, New Jersey, USA; 10 Institute of Public Health and Clinical Nutrition, University of Eastern Finland, Kuopio, Finland; 11 National Center for Environmental Assessment, U.S. Environmental Protection Agency, Cincinnati, Ohio, USA

**Keywords:** cardiovascular, dose-response function, health impact analysis, mercury, methylmercury, myocardial infarction

## Abstract

**Background:**

The U.S. Environmental Protection Agency (U.S. EPA) has estimated the neurological benefits of reductions in prenatal methylmercury (MeHg) exposure in past assessments of rules controlling mercury (Hg) emissions. A growing body of evidence suggests that MeHg exposure can also lead to increased risks of adverse cardiovascular impacts in exposed populations.

**Data extraction:**

The U.S. EPA assembled the authors of this article to participate in a workshop, where we reviewed the current science concerning cardiovascular health effects of MeHg exposure via fish and seafood consumption and provided recommendations concerning whether cardiovascular health effects should be included in future Hg regulatory impact analyses.

**Data synthesis:**

We found the body of evidence exploring the link between MeHg and acute myocardial infarction (MI) to be sufficiently strong to support its inclusion in future benefits analyses, based both on direct epidemiological evidence of an MeHg–MI link and on MeHg’s association with intermediary impacts that contribute to MI risk. Although additional research in this area would be beneficial to further clarify key characteristics of this relationship and the biological mechanisms that underlie it, we consider the current epidemiological literature sufficiently robust to support the development of a dose–response function.

**Conclusions:**

We recommend the development of a dose–response function relating MeHg exposures with MIs for use in regulatory benefits analyses of future rules targeting Hg air emissions.

Methylmercury (MeHg) is a widespread and particularly toxic form of mercury (Hg). It results from the conversion of inorganic Hg to a methylated form by aquatic microorganisms and can bioaccumulate in the aquatic food web. Dietary intake of MeHg, primarily through ingestion of contaminated fish and seafood, is recognized as a significant public health concern, primarily because of its well-studied neurodevelopmental toxicity in fetuses and children. However, a growing body of evidence suggests that MeHg exposure may also lead to increased risks of adverse cardiovascular impacts in exposed populations.

In a comprehensive review of MeHg-related health effects in 2000, the National Research Council (NRC) concluded that neurodevelopmental impacts from prenatal MeHg exposures are the most sensitive and best-documented end points (NRC 2000). The report also found limited evidence of adverse cardiovascular effects at similar levels of exposure but did not reach firm conclusions on the cardiovascular impact of MeHg intake.

Since the publication of the NRC report, the U.S. Environmental Protection Agency (EPA) benefits assessments of rules controlling Hg emissions, such as the Clean Air Mercury Rule, have quantified neurodevelopmental benefits of reducing MeHg exposures to fetuses and children (U.S. EPA 2005). [For a diagram outlining the U.S. EPA’s benefits assessment process, see Supplemental Material, Figure 1 (doi:10.1289/ehp.1003012).] However, Rice et al. (2010) developed a probabilistic analysis that characterized the plausible distribution of health and economic benefits associated with a reduction in MeHg exposure and reported that 80% of the benefits were associated with reductions in fatal heart attacks, and the remainder with IQ gains. Therefore, omitting these effects, if real, could result in a significant downward bias on the economic value of benefits ascribed to rules that control Hg emissions. Other assessments have reviewed the evidence for cardiovascular risk from MeHg exposure (Mozaffarian 2009; Stern 2005) and the balance of cardiovascular risks and benefits from MeHg exposure in conjunction with fish intake (Mozaffarian and Rimm 2006). However, previous assessments have not addressed the full range of potential cardiovascular health effects and have not focused on the development of dose–response relationships between MeHg and these individual cardiovascular effects.

In January 2010, the U.S. EPA convened a workshop in Washington, DC, to review the current science concerning cardiovascular impacts of MeHg exposures and to elicit recommendations about whether these effects should be included in benefits assessments of future Hg rules [for a list of questions posed by the U.S. EPA to workshop participants, see Supplemental Material (doi:10.1289/ehp.1003012)]. The invited panel consisted of nine individuals, all coauthors of this article, with expertise spanning epidemiology, clinical medicine, toxicology, risk and exposure assessment, biostatistics, and uncertainty analysis.

This article discusses the current literature and presents the recommendations of the assembled panel. In brief, we recommend the development of a dose–response function relating MeHg exposures with myocardial infarction (MI), for use in regulatory benefits analyses of future rules targeting Hg emissions.

## Background

### Hg fate and transport

Hg is present in most natural materials, including soils, coal, and minerals. It is released to the earth’s atmosphere naturally from crustal materials, including volcanic action, and from anthropogenic processes such as combustion of coal, waste combustion, and smelting. During atmospheric transport, chemical reactions slowly convert elemental Hg to divalent forms that can bind to particulate matter. Both divalent Hg and particulate Hg are deposited from the atmosphere onto water bodies, where microorganisms and chemical processes can convert a small proportion of the Hg to MeHg, which then bioaccumulates in fish. The most common route of MeHg exposure in humans is the consumption of marine and freshwater fish (Swain et al. 2007; U.S. EPA 1997).

### Measuring Hg exposure

Human MeHg exposures are often assessed through the use of biomarkers, which serve as a surrogate for the biologically relevant internal dose of MeHg (NRC 2000). Three main biomarkers are commonly reported in the literature to assess MeHg exposures: blood Hg, hair Hg, and toenail Hg.

Whole-blood Hg is more closely correlated with ingested dose than is hair Hg, the other commonly employed biomarker of MeHg exposure (Budtz-Jørgensen et al. 2004), and generally reflects short-term exposures [the average half-life of MeHg in blood is 50 days (NRC 2000)]. Blood Hg concentrations measured at a specific point in time reflect contributions from both recent exposures, which may be increasing if intake is ongoing, and older exposures whose relative contribution decreases over time. In populations with frequent, regular patterns of fish consumption, blood Hg can reflect a steady-state concentration and therefore could be an accurate measure of average intake over time (NRC 2000). In populations with infrequent or irregular fish consumption, this steady-state assumption may be inappropriate, and a single measure of blood may not provide enough information to assess the magnitude or timing of the exposures that contribute to the total blood Hg concentration.

Although whole-blood Hg is well correlated with MeHg exposure among populations who regularly consume fish, whole-blood Hg also reflects inorganic Hg, some of which arises from dental amalgams and some from demethylation of MeHg. MeHg is preferentially bound in the red blood cells (RBCs), whereas inorganic Hg distributes more evenly between RBCs and plasma. Thus, RBC Hg can be a more precise biomarker for MeHg, especially in populations with dental amalgams and low MeHg exposure (Berglund et al. 2005). However, RBC Hg has been less commonly reported than whole-blood Hg, and intake–biomarker relationships using RBC Hg have not been extensively investigated.

Hair Hg can provide some indication of both the magnitude and timing of exposures. The incorporation of Hg into the growing hair follicle is assumed to be directly proportional to the blood concentration, with a delay of 1–2 months between MeHg intake and measurement in the visible proximal hair shaft (Budtz-Jørgensen et al. 2004; NRC 2000). The relationship between location along the hair strand and timing of exposure can be ascertained by assuming a constant rate of hair growth; for example, a growth rate of 1.1 cm per month is commonly assumed (NRC 2000). Limitations of hair as an Hg biomarker include interindividual variability in the pharmacokinetics of Hg uptake from blood to hair shaft, inter- and intraindividual variability in hair growth rate, impact of hair treatment on hair Hg levels extraneous to the proximal end of the hair shaft, and the fact that the hair compartment is kinetically distant from ingestion and the target organ compartment (NRC 2000; Ohba et al. 2008).

Toenail Hg can be an indicator of average exposure over time (Rees et al. 2007). Garland et al. (1993) analyzed toenail Hg from two sets of clippings that were taken 6 years apart and reported a high level of reproducibility, which suggests that this biomarker can be representative of long-term exposure. Toenail clippings represent several weeks of nail growth that correspond to intake over the previous 3–12 months (Ohno et al. 2007; Rees et al. 2007). However, the specific toxicokinetics of Hg uptake from the blood to the nail bed are not well understood. In addition, toenails suffer from some of the same shortcomings as hair (i.e., kinetically distant from ingestion and from the target organ, as well as potential interindividual variability in nail growth).

## Workshop Findings and Near-Term Recommendations

The consensus finding of the workshop participants in support of an MeHg–MI dose–response function was based on a thorough consideration of available evidence, as discussed below. We have also developed a number of detailed recommendations that could be implemented in the near term to facilitate the development of a dose–response function.

### Likelihood of a causal relationship

We assessed the causal relationship between MeHg exposure and an increased risk of cardiovascular health effects by evaluating the plausibility of biological mechanisms for the cardiovascular toxicity of MeHg and weighing the strength of the human, animal, and *in vitro* studies linking MeHg with cardiovascular health impacts. Table 1 summarizes our assessment of the strength of the evidence presented in the literature evaluating MeHg and several cardiovascular health effects. We used professional judgment in our qualitative evaluations of the strength of evidence associated with each effect. Some of these effects represent clinical outcomes (e.g., hypertension and MI), whereas others are intermediary effects that may contribute to the progression of heart disease [e.g., oxidative stress, atherosclerosis, and decreased heart rate variability (HRV)]. Our examination of the evidence linking MeHg with these intermediary effects, as described below, provides additional information to evaluate the plausibility of the associations between MeHg and clinical cardiovascular disease.

#### Oxidative stress

MeHg exposure can cause oxidative stress, which is an early biological response that can produce vascular endothelial cell damage by promoting inflammation and vasoconstriction as well as lipid peroxidation through the generation of reactive oxygen species (ROS) (Grotto et al. 2009). Oxidative stress can lead to the development of cardiovascular disease by contributing to arrhythmias, hypertension, and atherosclerotic plaque development and rupture (Briones and Touyz 2010; Dzau et al. 2006; Van Wagoner 2008).

Multiple hypotheses concerning the ability of MeHg to induce oxidative stress have been proposed (Salonen et al. 1995). These involve either direct effects of MeHg, where it catalyzes reactions that produce ROS, possibly generated during its metabolism to inorganic Hg, or indirect effects of MeHg, where it binds to sulfhydryl groups or selenium and inactivates antioxidant thiol compounds or peroxide-scavenging enzymes such as glutathione peroxidase (Grotto et al. 2009; Khan and Wang 2009).

Two epidemiological studies support a link between MeHg exposure and oxidative stress. Salonen et al. (1995) reported elevated MeHg levels associated with increased levels of oxidized low-density lipoprotein (LDL) in serum from a subset of a cohort of Finnish men. In a study of Amazonian communities with high fish consumption, Grotto et al. (2010) found significant associations between biomarkers of MeHg exposure (blood Hg and hair Hg) and markers of oxidative stress (e.g., glutathione, glutathione peroxidase, and catalase). However, a study conducted in Quebec, Canada, before and after the freshwater sport fishing season in James Bay showed that oxidized LDL decreased by about 20% and antioxidants increased slightly in fishermen, despite a doubling of hair Hg levels over the same period (Bélanger et al. 2008).

Animal studies have consistently found evidence for MeHg-induced oxidative stress. For instance, MeHg has been shown to increase lipid peroxidation in rats (Huang et al. 1996; Lin et al. 1996). In addition, Grotto et al. (2009) found that long-term, low-dose exposure to MeHg increased production of ROS in rats as inferred from increased production of malondialdehyde (a secondary product of lipid peroxidation and a biomarker of cell membrane injury), depletion of glutathione, and depressed nitrogen oxide bioavailability (a marker of endothelial dysfunction).

*In vitro* evidence mostly supports an indirect mechanism for MeHg-related oxidative stress. Gatti et al. (2004) found MeHg toxicity *in vitro* via glutathione depletion, with little evidence of increased ROS. Similarly, Seppänen et al. (2004) found no evidence for promotion of direct lipid peroxidation by MeHg. Other *in vitro* studies have provided support for the hypothesis that MeHg can interfere with signal transduction and proper endothelial cell function [both in human umbilical cord endothelial cells (Hagele et al. 2007; Mazerik et al. 2007; Peltz et al. 2009) and bovine endothelial cells (Kishimoto et al. 1995; Ohno et al. 1995)], which may influence atherosclerosis. However, three studies support a direct mechanism for MeHg’s role in oxidation, showing that antioxidants and compounds that remove ROS can mitigate MeHg toxicity (Gasso et al. 2001; Park et al. 1996; Welsh 1979).

Although the specific mechanism for MeHg’s role in oxidative stress is uncertain, we found the evidence for MeHg-induced oxidation to be “moderate to strong,” based on the strength of the available epidemiological and animal studies.

#### Atherosclerosis

Most acute MIs are caused by the blockage of a coronary artery, leading to ischemia and heart cell death. Atherosclerosis can directly limit blood flow in these arteries through significant plaque buildup and release of vasoconstrictors (Vander et al. 2001). Blood clots can also form in the plaque, detach, and lead to a blockage.

One possible pathway for MeHg’s effects on atherosclerosis is through oxidative stress, as described above. Another possibility explored through *in vitro* studies is that MeHg influences adhesion molecules, which promote atherosclerosis by attracting white blood cells to the endothelium of the artery (Prozialeck et al. 2002).

Epidemiological studies that have explored a link between MeHg and atherosclerosis generally focus on the intima-media thickness (IMT) of the carotid artery, a common measure for assessing the progression of atherosclerosis (Stein et al. 2008). Three epidemiological studies support a link between MeHg exposure and increased IMT. The first, a study of Faroese whaling men, found that MeHg exposure biomarkers were significantly associated with increased IMT, with toenail Hg being the best predictor (Choi et al. 2009). In a study of Finnish men, those in the highest quintile of hair Hg exhibited a 32% increase in mean carotid IMT compared with those in all other quintiles (Salonen et al. 2000). IMT also increased with increasing Hg levels among Nunavik Inuit adults in Quebec (Dewailly et al. 2009). However, in a fourth study of a cohort of Indian Cree adults, Hg was not associated with changes in IMT (Viger et al. 2007).

Based on the plausibility of the mechanisms for the effects of MeHg on atherosclerosis and the generally supportive epidemiological evidence, we classify the strength of the IMT end point as “moderate.”

#### Heart rate variability

HRV is an indicator of the dynamic adaptation capacity of the cardiac system and is a measure that is commonly used in epidemiological studies to evaluate autonomic nervous function. Decreased HRV is a common feature of cardiovascular disease and predicts increased risk of cardiovascular mortality, particularly in the elderly (Hattis 2003; Lahiri et al. 2008). It is possible that this mechanism works through MeHg toxicity to the neurological system, because autonomic function is governed by the central nervous system; however, specific evidence of this mechanism is lacking at the dose levels of interest.

Existing epidemiological literature consistently shows a decrease in HRV with MeHg exposure, although the specific measures of HRV in each study vary. In a recent intervention study, Yaginuma-Sakurai et al. (2009), randomly assigned a sample of healthy Japanese adults to either an experimental group, in which the participants were exposed to MeHg for 14 weeks at Japan’s provisional tolerable weekly intake for adults by consuming appropriately sized meals of tuna and swordfish, or to a control group. In the experimental group, hair Hg increased by nearly a factor of 4, and HRV (specifically the sympathovagal balance index) decreased significantly. After a 15-week “washout” period, HRV measurements in the experimental group returned to baseline. These results suggest that moderate dietary intake of MeHg over relatively short periods can induce sympathodominant states (reflecting either reduced vagal or elevated sympathetic activity) that reduce HRV. However, the very long half-life of MeHg observed in this study (mean = 105 days) makes interpretation of this study somewhat uncertain.

HRV decreased with increasing MeHg in a study of Nunavik Inuit adults in Canada (Valera et al. 2008). Studies of Cree Indians and Polynesian populations also showed similar results (Valera et al. 2009a, 2010). In a study of those living near an industrial complex in Korea, HRV decreased > 8% per 1-ppm increase in hair Hg levels (Lim et al. 2009). However, the Faroese whaling study by Choi et al. (2009) reported that the effects of MeHg on HRV were equivocal.

These associations have also been observed in children (Grandjean et al. 2004; Murata et al. 2006; Sørensen et al. 1999), although the direct significance of decreased HRV in young children for later development of cardiovascular disease is unknown.

Although the specific mechanism for the effects of MeHg on HRV has not been elucidated, we found that the epidemiological literature is generally consistent in reporting an association between MeHg and HRV, which led us to classify the evidence supporting this end point as “strong.”

#### Blood pressure and hypertension

Increased blood pressure (BP) or hypertension can enhance the development of atherosclerosis and MI. In addition, hypertension is a cardiovascular health end point in and of itself that can also lead to other health problems such as heart failure, kidney damage, or stroke (Lawes et al. 2008).

Several studies have found evidence of a link between MeHg and increased measures of BP, although the specific measure varies from study to study. Two studies found a positive association between MeHg exposure and systolic blood pressure (SBP): a study of Inuit adults (Valera et al. 2009b) and a study of individuals from the Brazilian Amazon (Fillion et al. 2006). In addition, two studies report positive associations between MeHg exposure and increased diastolic blood pressure (DBP): the Faroese whaling men study by Choi et al. (2009) and a study in a Philippine gold mining community (Cortes-Maramba et al. 2006). However, the latter study may include Hg intake via routes other than fish and through forms of Hg other than MeHg (e.g., documented storage of elemental Hg in homes). Results from studies in French Polynesia were suggestive of a positive association with both SBP and DBP, but the association was not significant (Valera et al. 2009a). Finally, a study of 24-hr ambulatory BP in samples of Danes and Greenlanders found that blood Hg was positively correlated with pulse pressure only (Pedersen et al. 2005).

MeHg and BP associations have also been identified in children. In the Faroese cohort, Sørensen et al. (1999) observed associations among children between increased SBP and DBP and prenatal MeHg exposure at the 7-year evaluation. A study of children from the Seychelles Child Development Study, who were tested at 12 and 15 years of age, found increased DBP in 15-year-old boys prenatally exposed to MeHg (Thurston et al. 2007).

Studies that did not find increased BP with increased MeHg included another study of Amazonian Indians (Dórea et al. 2005) that found a trend of smaller increases in BP with age among higher fish consumers. A study of Saudi Arabian women (Al-Saleh et al. 2006) reported no association between hypertension and Hg; however, this study used serum Hg as a biomarker, which largely reflects inorganic Hg exposure. In addition, the previously observed effect on BP was not found in children of the Faroese cohort at the 14-year follow-up (Grandjean et al. 2004).

In animal studies, long-term exposure to a low dose of MeHg (100 μg/kg/day) increased SBP in Wistar rats (Grotto et al. 2009); these results are consistent with the results of other studies in rats that administered higher doses (Tamashiro et al. 1986; Wakita 1987).

Because of the inconsistency in the results of the epidemiological studies in terms of the specific BP measure affected in humans and whether a link was found with MeHg exposure, we found the evidence for MeHg’s effect on BP to be “weak.”

#### Myocardial infarction

Four major epidemiological studies published in the last decade have examined the direct association between MeHg exposure through fish consumption and risk of MI: the European Community Multicenter Study on Antioxidants, Myocardial Infarction and Breast Cancer (EURAMIC; Guallar et al. 2002), the Kuopio Ischaemic Heart Disease Risk Factor (KIHD) study (Virtanen et al. 2005), the Health Professionals Follow-up Study (HPFS; Yoshizawa et al. 2002), and the Northern Sweden Health and Disease Study (NSHDS; Hallgren et al. 2001). Table 2 gives a detailed summary of these key studies.

EURAMIC and the KIHD study both report significant positive associations between MeHg exposure and MI incidence. Strengths of these two studies include their relatively large sample sizes, control for a number of key potential confounders, and inclusion of only first MI, reducing the likelihood that patients had changed their diet before the event. Additional strengths of EURAMIC include exposure data that were collected shortly after the MI, reducing the possibility that the measurements were affected by the development of disease, and recruitment of participants across eight European countries and Israel, resulting in a wide range of MeHg and fish oil intake (Guallar et al. 2002). Additional strengths of the KIHD study include its prospective design, population-based recruitment, homogeneous study population, limited loss to follow-up, and a diet of mostly nonfatty freshwater fish, which reduces the possibility of confounding by cardioprotective compounds that are correlated with fat composition of fish.

The HPFS did not find an association between MeHg and MI when analyzing the entire study population. However, the HPFS measured fatty acid intake through a questionnaire on fish consumption, which may lead to residual confounding due to the potential inaccuracies of the semiquantitative food frequency questionnaire as opposed to direct measurement. In addition, the results could be confounded by occupational inorganic Hg exposure in dentists, who comprise almost half of the study population. Excluding dentists from the analysis resulted in an elevated, although nonsignificant, association (Table 2). Yoshizawa et al. (2002) note that this supplemental analysis included a smaller number of cases (220) and therefore lacks sufficient statistical power to detect an effect.

In the NSHDS, Hallgren et al. (2001) reported an inverse association between MeHg and MI. However, this study suffers from small sample size (78 cases), the potential for exposure misclassification [for example, a single measurement of erythrocyte Hg (Ery-Hg) taken at enrollment—up to 10 years before the MI—may not accurately represent long-term exposures of concern], and an exposure gradient that is potentially too narrow to detect an association. Only two subjects in this study had Ery-Hg levels that corresponded to hair Hg levels > 2 μg/g, the level above which the KIHD study found positive effects (Virtanen et al. 2007).

**Figure 1:**
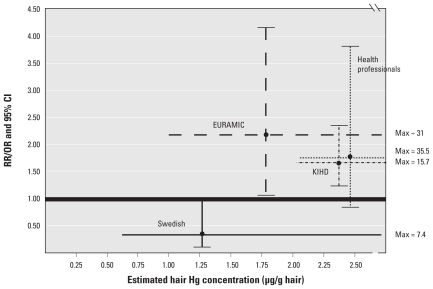
Consistency and strength of association for Hg–cardiovascular risks: RRs and odds ratios (ORs). Abbreviations: CI, confidence interval; Max, maximum hair Hg. Adapted from Rice et al. (2010). “Swedish” results refer to NSHDS. The plot of the HPFS (“Health Professionals”) represents the results of a separate multivariate analysis (*n* = 220) excluding subjects likely exposed occupationally to inorganic Hg (i.e., dentists). To convert toenail Hg levels reported in EURAMIC and HPFS to hair Hg levels, Rice et al. (2010) used the regression model developed by Ohno et al. (2007) from an analysis excluding women with artificial hair waving. To convert the Ery-Hg concentrations reported in NSHDS to hair Hg, Rice et al. (2010) first estimated the corresponding Hg levels expected in whole blood by multiplying the concentration of Hg in the erythrocytes by the specific gravity of erythrocytes (1,093 g/L) and the average male hematocrit (46%). They then used data from the World Health Organization that measured the distribution of MeHg between human erythrocytes and plasma (20:1) and a blood:hair partition estimate (Allen et al. 2007; Shipp et al. 2000).

Overall, we believe the link between MeHg and MI is plausible, given the range of intermediary effects for which some positive evidence exists and the strength and consistency across the epidemiological studies for MI. Figure 1 illustrates this consistency, showing significant overlap between the effect estimates from the four major studies of MI. Three of the four studies have relative risks (RRs) that are elevated and somewhat clustered (these RRs fall between ~ 1.5 and 2.0), with overlapping confidence bounds. However, some uncertainty remains as to whether a causal relationship exists because of ambiguity in the specific biological mechanism involved, the small number of epidemiological studies, and inconsistencies in the literature supporting some of the intermediary effects (e.g., BP). Therefore, as a group, we found the evidence of MI to be “moderate to strong.” During the workshop, the individual authors provided quantitative estimates of the likelihood of a true causal relationship between MeHg and MI that ranged from 0.45 to 0.80.

### Recommended cardiovascular end points to include in benefits assessments

In making recommendations on cardiovascular end points for inclusion in an MeHg benefits assessment, we considered both the strength of evidence linking the outcome with MeHg and whether it has sufficient clinical specificity that changes in incidence could be estimated and economic valuation could be applied. We considered two of the five cardiovascular end points to satisfy the latter condition: hypertension and acute MI. However, of the two, we believe that only MI has sufficient evidence to support its inclusion in a benefits assessment, based on both the current evidence linking MeHg to MI directly and the influence of MeHg on intermediary effects leading to MI. Therefore, we recommend that future benefits analyses of MeHg reductions include a quantification of avoided cases of MI in adults.

### Other recommendations for developing a dose–response function

Below, we provide recommendations on key factors relating to the derivation of a dose–response function relating MeHg exposure with MI.

#### Epidemiological evidence

Of the four main studies that examined the MeHg–MI relationship, we believe EURAMIC and the KIHD study provide the strongest and most useful data sets for quantifying MeHg-related incidence of MI for benefits assessment because of their strengths outlined above. The methodological issues associated with the HPFS and NSHDS, discussed in the preceding section, limit the usefulness of these studies for deriving a dose–response function for benefits assessment.

#### Exposure metric

For the purposes of an MeHg regulatory benefits analysis centered on the MI outcome, where the toxicity of MeHg is likely to operate over long periods of exposure, the use of hair Hg or toenail Hg may prove to be more useful than blood Hg. These two biomarkers allow for an assessment of a longer-term average Hg concentration that may be more pertinent for mechanisms leading to MI (e.g., increased IMT over time).

#### Shape of the dose–response function

**Figure 2:**
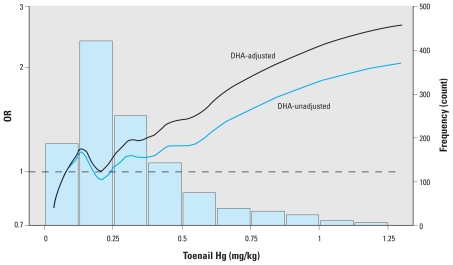
Nonparametric odds ratios (ORs) of MI by level of toenail Hg from EURAMIC. DHA, docosahexaenoic acid. The right-hand *y*-axis indicates the number of subjects in each bin of toenail Hg concentration. From Guallar et al. (2002);. reproduced with permission from the *New England Journal of Medicine*, Massachusetts Medical Society (2002). All rights reserved.

Many of the existing MI epidemiological studies include Hg biomarker concentration as a categorical variable (e.g., quintiles of biomarker concentration). However, EURAMIC used generalized additive models (GAMs) to help elucidate the shape of the dose–response function (Guallar et al. 2002; Hastie and Tibshirani 1990). This analysis suggested a roughly linear relationship between log-transformed toenail Hg concentrations and the log odds of MI (see Figure 2). Reanalysis of the KIHD data set using flexible dose–response models such as GAM or spline regression models would provide a consistent basis for the evaluation of the shape of the dose–response function across studies [for further information on GAMs, see Supplemental Material (doi:10.1289/ehp.1003012)].

#### Confounding and effect modification

One important factor to consider when deriving a dose–response function for the MeHg–MI relationship is the potential for “negative confounding” by the presence of n-3 polyunsaturated fatty acids (PUFAs) in the fish consumed (i.e., docosapentaenoic, docosahexaenoic, and eicosapentaenoic acids). Negative confounding refers to a factor resulting in an underestimate of the true effect if not properly controlled (Choi et al. 2008). PUFAs are found largely in oily, cold-water fish, such as salmon and herring (Mahaffey et al. 2008). These compounds have been linked to a decreased risk of cardiovascular disease (Mozaffarian and Rimm 2006). Therefore, it is important that the coefficient for an MeHg–MI dose–response function be derived from a model that fully controls for confounding by PUFAs, in order to avoid introducing bias into the estimates.

To date, existing epidemiological studies have controlled for this confounder by assuming a linear relationship between PUFAs and risk of MI. However, the association between PUFA intake and cardiovascular risk may not be linear (Mozaffarian and Rimm 2006). Therefore, going forward, we recommend reanalysis of the epidemiological data to incorporate nonlinear adjustments for this negative confounder in order to reduce the possibility of an artificially attenuated dose–response coefficient [for more information, see Supplemental Material (doi:10.1289/ehp.1003012)].

Another consideration in the derivation of the dose–response function is possible effect modification by cardioprotective compounds. We found mixed results in epidemiological studies that assessed the possibility of effect modification through the use of interaction terms. EURAMIC and HPFS do not support effect modification by PUFAs or selenium, respectively, whereas the KIHD study did find a statistically significant interaction term between MeHg exposure and PUFAs. At this time, the evidence is insufficient to conclude whether effect modification by cardioprotective compounds occurs, because of the inconsistent results across the epidemiological studies and the lack of mechanistic evidence. In addition, differentiating between effect modification by cardioprotective compounds and a nonlinear dose–response function can be difficult when exposure is moderately to highly correlated with a confounder.

#### Definition of outcome

Another key question is whether separate dose–response functions can or should be estimated for fatal and nonfatal MIs. Three of the epidemiological studies—KIHD, HPFS, and NSHDS—provide risk estimates for total MI, including both fatal and nonfatal outcomes. EURAMIC provides a RR for nonfatal MIs. At this time, we believe there is insufficient evidence to construct separate dose–response functions for fatal and nonfatal MIs. Therefore, we recommend that a single dose–response function from the existing literature be derived that would represent total MIs. Data on the survivability of MIs could then be used to determine the fraction of avoided MeHg-related MIs that would be fatal versus nonfatal in a benefits assessment.

#### Pooling across studies

When deriving an MeHg–MI dose–response function, it may be useful to pool across the two recommended studies, EURAMIC and KIHD, to increase statistical power and widen the range of exposure levels to which the dose–response function could be applied. One way to pool the data from these two studies would be to convert the exposure data to a single biomarker. This can be achieved through the use of biomarker ratios. If the assumption is made that MeHg consumption occurs with a sufficiently high frequency over a long period of time to allow the body burden of MeHg to reach approximate steady state, it is possible to derive hair-to-blood, hair-to-toenail, or toenail-to-blood ratios (Bartell et al. 2000). Multiple studies have attempted to calculate these ratios (e.g., Bjorkman et al. 2007; Choi et al. 2009; Ohno et al. 2007). However, a large amount of variability and uncertainty is associated with these values, stemming from the significant interindividual variability in toxicokinetics of MeHg (NRC 2000), evidence of laboratory measurement error (Bartell et al. 2000), and uncertainty about the assumption of steady-state conditions and the temporal association between exposure and Hg measurement (i.e., blood and hair samples obtained at the same time do not yield Hg exposure values that reflect identical exposure time periods).

Another approach would be to assume that the log of the odds of an MI is, over the range of exposures in the general population, proportional to the logarithm of MeHg exposure. If this is true, and these models were refit for the two recommended studies, then the coefficients would be interpretable as the percent change in the odds of an MI for a given percentage change in the exposure metric. However, it would be necessary to explore whether there is biological justification for altering the functional form of the relationship between exposure and effect in this way. It may be worthwhile to analyze the data using both of these approaches to help characterize uncertainty in the analysis, because each method is subject to different sources of error.

To determine whether pooling of these two studies is reasonable, we examined the Hg levels of the study populations in these two studies by converting them to the same biomarker using a steady-state ratio from Ohno et al. (2007) (2.44 μg Hg/g hair per μg Hg/g toenail). The results of this conversion lead us to believe that the exposure levels across the two groups of study participants cover a range of values along the distribution of MeHg exposure in adults, and therefore it is reasonable to pool the results. [The mean hair Hg in the KIHD study is 1.9 μg/g compared with a mean of 0.66 μg/g in EURAMIC, with the individual study centers in that study ranging from 0.34 to 1.66 μg/g; for further details, see Supplemental Materials (doi:10.1289/ehp.1003012).]

### Recommendations for applying the dose–response function in benefits assessment

The current MeHg–MI epidemiological studies include mostly middle-age and elderly men. Therefore, it is uncertain whether a dose–response function derived for this subpopulation would be generalizable to women. Despite the limited number of studies of MeHg and MI in women, there is an overwhelming body of cardiovascular research in which risk factors for cardiovascular disease are largely similar for men and women, with high generalizability of findings between sexes (Mosca et al. 2004). Thus, it appears plausible that MeHg-related MIs could occur in women as well as men. Therefore, we recommend that a dose–response function derived from the current epidemiological literature be applied to both men and women in a benefits assessment. However, the size of the impact in women would be considerably more uncertain than that found in men, given the paucity of the direct empirical data.

Another important consideration for benefits assessment is whether the exposure levels experienced by the populations in the recommended epidemiological studies are comparable with exposure levels in general U.S. population. We found that the hair Hg levels in EURAMIC and the KIHD study correspond to the upper end of the distribution of hair Hg in women of childbearing age in the United States [~ 75th–95th percentile; for further detail, see Supplemental Material (doi:10.1289/ehp.1003012)]. Therefore, consideration could be given to restricting application of the dose–response function derived from these studies to only those with higher end exposures (i.e., include a possible threshold for MeHg toxicity). However, there is some evidence supporting a log-linear dose–response function (e.g., the GAM analysis in EURAMIC). If the relationship is in fact log-linear, it would be appropriate to apply a single slope across all levels of MeHg exposure.

## Future Research Needs

There is a need to expand the literature base describing the cardiovascular effects of MeHg. The workshop highlighted the following specific priorities.

### Additional epidemiological studies

Additional epidemiological studies of the relationship between MeHg biomarkers and MI are critical for helping to address whether there is a causal relationship underlying these reported associations. Such studies could also reduce uncertainties related to quantifying the MeHg–MI dose–response function.

### Dose–response assessment

A primary concern for reducing uncertainty in the application of an MeHg–MI dose–response function to the general U.S. population is the need to expand the database of the effects of MeHg levels on MI risk in general, especially in women, in minorities, and in patients who have preexisting cardiovascular disease. Additionally, we recommend further research into possible effect modification of the MI effect by selenium and by diabetes status, the latter of which can evaluate whether persons with diabetes represent a sensitive subpopulation.

### Research on mechanisms

Available studies do not yet provide a clear picture of the biological mechanisms underlying the cardiovascular effects observed in toxicological and epidemiological studies.

### Exposure assessment

There is a need to identify existing data sets or collect and analyze new data that measure multiple biomarkers of Hg in individuals concurrently and repeatedly over the course of a year or longer. These data will help to better characterize or reduce exposure misclassification resulting from intraindividual variability over time in individual biomarkers. They will also improve the scientific understanding of the relationships between different biomarkers and help to reduce uncertainty in biomarker ratios. Future epidemiological studies would greatly benefit by incorporating multiple biomarkers. This would help refine dose–response relationships by allowing the use of the exposure metric that provided the most robust statistical outcome, and by permitting the use of approaches that integrate these multiple measures such as structural equation models [for further detail, see Supplemental Materials doi:10.1289/ehp.1003012)].

### Timing of benefits

At this time, little is known about how quickly MI risk or other cardiovascular risk factors may decrease after reductions in MeHg exposure. Yaginuma-Sakurai et al. (2009) suggest that recovery of HRV from short-term spikes is relatively quick, but similar types of intervention studies that explore the effects of reductions in MeHg intake on cardiovascular risk factors in chronic consumers of high MeHg fish species would be particularly informative for benefits analyses.

### Other cardiovascular end points

Currently, there is a lack of studies examining a possible link between MeHg exposures and risk of sudden cardiac death, peripheral arterial disease, and heart failure and very limited data on the link between MeHg exposure and stroke (Wennberg et al. 2007), all of which are related to some of the risk factors examined in this article (e.g., hypertension and atherosclerosis). Therefore, these are important end points to include in future studies on the cardiovascular effects of MeHg.

## Figures and Tables

**Table 1:** Assessment of the biological plausibility of MeHg-related MI.

	Epidemiological		Animal		*In vitro*		
Effects	No. of studies	Strength of evidence	No. of studies	Strength of evidence	No. of studies	Strength of evidence	Overall
Oxidation	Few	Weak to moderate	Few	Moderate to strong	Some	Weak	Moderate to strong
Atherosclerosis/IMT	Some	Moderate	None	NA	None	NA	Moderate
HRV	Some	Strong	NA	NA	NA	NA	Strong
BP/hypertension	Some	Weak to moderate	Few	Weak	NA	NA	Weak
Fatal and nonfatal MI	Some	Moderate	None	NA	NA	NA	Moderate
Biological plausibility for MI considering intermediary effects							Moderate to strong

Abbreviations: BP, blood pressure; Few, 1–3 studies; IMT, intima-media thickness; MI, myocardial infarction. NA, not applicable; Some, 4–12 studies. Workshop participants used professional judgment to qualitatively evaluate the strength of evidence associated with each effect.

**Table 2:** Summary of the epidemiological literature on the effects of MeHg on the incidence of MI.

Study	Location	Study population	Sample size	Outcomes included	Hg biomarker	Fatty acid measure	Results
EURAMIC case–control study (Guallar et al. 2002)	Eight European cites and Israel	Men ≤ 70 years of age (mean of cases = 54.7 years); retrospective design	684 cases, 724 controls	First nonfatal MI	Toenail	DHA in adipose tissue	After controlling for DHA and a number of risk factors for heart disease, the authors reported an OR of 2.16 (95% CI, 1.09–4.29; *p*-value for trend = 0.006) comparing the highest quintile of toenail Hg level with the lowest. A positive monotonic increase in the risk of MI exists with toenail Hg content > 0.25 μg/g. Slope increases with adjustment for adipose tissue DHA content. The OR associated with a change from the 25th to the 75th percentile of toenail Hg was 1.63 (95% CI, 1.22–2.18).
KIHD cohort study (Virtanen et al. 2005)	Eastern Finland	Men 42, 48, 54, or 60 years of age at baseline examination; prospective design	1,871 (282 cases)	First acute coronary event^a^	Hair	DHA and DPA in serum	After controlling for several cardiovascular disease risk factors and DHA and DPA levels, men in the highest third of hair Hg content had a 1.60-fold (95% CI, 1.24–2.06) increased risk of an acute coronary event, compared with the two lower thirds combined. On average, the risk of an acute coronary event increased by 11% for each 1-μg/g increase in hair Hg concentration.
HPFS nested case–control study (Yoshizawa et al. 2002)	USA	Male health professionals 40–75 years of age at baseline; prospective design	470 cases, 464 controls	CHD (fatal CHD, nonfatal MI, and coronary revascularization)	Toenail	Dietary questionnaires assessing fish intake	In a multivariate analysis, the RR of nonfatal MI or fatal CHD for men in the highest versus the lowest quintile of toenail Hg level was 1.04 (95% CI, 0.65–1.68; *p*-value for trend = 0.68). An analysis excluding dentists found an elevated, although nonsignificant, association (RR = 1.70; 95% CI, 0.78–3.73; *p*-value for trend = 0.41). The authors note that this supplemental analysis included a smaller number of cases (220) and therefore is associated with reduced statistical power.
NSHDS nested case–control study (Hallgren et al. 2001)	Sweden	Men and women 30, 40, 50, or 60 years of age at enrollment; prospective design	78 cases, 156 controls	First MI (fatal or nonfatal)	Erythrocytes	EPNA and DHA in plasma phospholipids	Inverse association found between Ery-Hg concentration and risk of MI (RR = 0.51; 95% CI, 0.21–1.24) comparing high versus low Ery-Hg groups.

Abbreviations: CHD, coronary heart disease; CI, confidence interval; DHA, docosahexaenoic acid; DPA, docosapentaenoic acid; EPNA, eicosapentaenoic acid; ERY-Hg, erythrocyte Hg; OR, odds ratio.

a This included definite and probable MI and typical prolonged chest pain episodes.
